# Moral distress among maternal-fetal medicine fellows: a national survey study

**DOI:** 10.1186/s12910-025-01187-4

**Published:** 2025-02-28

**Authors:** Jia Jennifer Ding, Thi Vu, Suzanne Stammler, Peter Murray, Elizabeth Epstein, Sarah N. Cross

**Affiliations:** 1 Division of Maternal-Fetal Medicine, Department of Obstetrics, Gynecology, and Reproductive Sciences, Yale School of Medicine, 333 Cedar Street, P.O.B. 208063, New Haven, Connecticut, CT 06520 United States of America; 2 Department of Social and Behavioral Sciences, Yale School of Public Health, New Haven, Connecticut, United States of America; 3 Department of Pediatrics-Neonatal/Perinatal, University of Virginia, Charlottesville, VA United States of America; 4 School of Nursing and Center for Health Humanities and Ethics, University of Virginia, Charlottesville, VA United States of America

**Keywords:** Moral distress, Maternal-fetal medicine fellows, Abortion restriction, Maternal mortality

## Abstract

**Background:**

Moral distress, or the inability to carry out what one believes to be ethically appropriate because of constraints or barriers, is understudied in obstetrics and gynecology. We sought to characterize moral distress among Maternal-Fetal Medicine (MFM) fellows using a standardized survey.

**Methods:**

We disseminated a national anonymized survey study of MFM fellows electronically regarding moral distress using a validated questionnaire with supplemental questions pertaining to specific challenges within MFM clinical care. Multivariable linear regression modeling was used to examine the association between abortion restrictions, maternal mortality, and moral distress, controlling for demographic variables. Thematic analysis was performed for the free text responses elaborating upon moral distress and grouped by thematic elements. We hypothesized that training in states with more abortion restrictions and higher maternal mortality would be associated with higher moral distress scores.

**Results:**

Among 245 total responses (61% response rate), 177 complete responses (44% complete response rate) were included for analysis. Most of our respondents identified as female (78.5%), White (71.8%), and training in urban programs (83.1%). 37.9% of respondents reported training in the Northeast, with the remainder of respondents evenly distributed across the United States. The mean score for the validated questions was 85.9 *±* 48.8, with female gender identity associated with higher measures of moral distress on the validated portion of the questionnaire as compared to male gender identity (90.1 *±* 49.2 vs. 70.4 *±* 44.7, *p* < 0.05), whereas more advanced training was associated with higher measures of moral distress on the supplemental questions as compared to those less advanced in training (20.9 *±* 11.8 vs. 28.5 *±* 15.9 vs. 25.9 *±* 15.6 for PGY-5 vs. PGY-6 vs. PGY-7 and PGY-8 combined, respectively, *p* < 0.05). After adjustment, higher measure of moral distress on the validated questionnaire was associated with training in states designated “Abortion restrictive” as compared to “Abortion most protective” (beta estimate 27.80 and *p* < 0.01). Of 34 free responses, 65% referred to limitations on abortion access and reproductive justice as causes of significant moral distress.

**Conclusion:**

MFM fellows who identify as female reported higher measures of moral distress, as well as those training in states with more abortion restrictions. Among free text respondents, abortion restrictions underlie a significant proportion of moral distress.

**Supplementary Information:**

The online version contains supplementary material available at 10.1186/s12910-025-01187-4.

## Background

Moral distress, or the inability to carry out what one believes to be ethically appropriate because of uncontrollable constraints or barriers, is understudied in obstetrics and gynecology (OB/GYN), and specifically in Maternal-Fetal Medicine (MFM). Originally identified in nursing, the concept of moral distress has since been studied among an array of healthcare professionals, including physicians, respiratory therapists, social workers, and healthcare organization administrators [1–9]. Moral distress can stem from factors such as challenges in clinical care, barriers in interdisciplinary collaboration, and systems inefficiencies. Moral distress can contribute to burnout and intention to leave one’s position in healthcare. The original validated survey, the Moral-Distress Survey-Revised (MDS-R) was re-evaluated recently and redesigned as the Measure of Moral Distress – Healthcare Professionals (MMD-HP) [1] which is specifically geared towards healthcare professionals. However, to our knowledge, moral distress using this validated tool has not been studied among OB/GYNs or MFMs, and not at a national level. Rising maternal mortality and increasing reproductive rights restrictions in large parts of the country would both likely impact OB/GYN job satisfaction and feelings of moral distress [10–12]. There is evidence already that OB/GYN trainees are avoiding pursuing training in states with reproductive restrictions [13], likely due to a combination of limitations around providing evidence-based care and concerns regarding legality of management of pregnancy complications [14]. Specifically, as MFM physicians are often the ones to diagnose and counsel regarding high-risk maternal, pregnancy, and fetal complications (such as cyanotic maternal cardiac disease, previable rupture of membranes, and severe fetal anomalies), restrictions on pregnancy management uniquely impact those within MFM. Therefore, we aim to establish a baseline measure of moral distress among MFM fellows, and to compare measures of moral distress between fellows who practice in regions with differing rates of maternal mortality and reproductive restrictions.

## Methods

We performed an anonymous cross-sectional national survey of MFM fellows in the United States using a validated survey tool, the Measure of Moral Distress – Healthcare Professionals (MMD-HP) (Supplemental materials 1). This validated survey describes 27 scenarios (i.e., “participate in care that causes unnecessary suffering or does not adequately relieve pain or symptoms” and “participate in care that I do not agree with but do so because of fears of litigation”) and asks participants to rate the *frequency* of scenario and *level of distress* per scenario, on a 5-point Likert scale (scored from 0 to 4). Scores are first multiplied across frequency and level of distress, then summed across the 27 questions. The total score can range from 0 to 432 points, with higher scores indicating an increase in moral distress. After the validated questions are addressed, surveys can be supplemented with additional specialty-specific scenarios to explore additional areas of moral distress. These supplemental questions however, do not contribute to the overall score. For this study, we created six supplemental scenarios describing the balance of maternal risk with fetal benefit, situations of medical uncertainty or futility, and allocation of resources (Supplemental materials 2). These supplemental questions were developed iteratively and internally within our own MFM division. The score for the supplemental section can range from 0 to 96 points. The survey also gathered basic demographic questions such as characteristics of the training program, religiosity, and political affiliation and included a free text field where respondents could optionally choose to elaborate on specific scenarios of moral distress.

This voluntary 15-minute survey was disseminated via electronic mail to all MFM fellows in the United States either via direct email or through program coordinators according to the Society for Maternal-Fetal Medicine Fellowship Directory. All MFM fellows in training, either in stand-alone MFM programs or combined programs (i.e., genetics, critical care) were invited to participate. In addition, the survey link was shared on various social media groups and listservs with MFM trainee members. We estimated a total of 400 MFM fellows based on program rosters and anticipated a response rate of 60%. We offered twelve randomly selected participants a twenty-five dollar e-gift card as incentive for completing the study. Only completed responses were analyzed. The survey was open from February 7, 2024 to May 5, 2024. Responses were captured and analyzed on the secure platform Qualtrics. Respondents were also grouped based on state of training according to abortion restrictions and maternal mortality rates. For abortion restriction, we referred to the Guttmacher Institute abortion access map (designations active as of May 2024) and collapsed their seven categories into four groups for ease of analysis [15]. States belonging to the Guttmacher Institute’s “most restrictive” and “very restrictive” categories were collapsed into a group we named “Abortion very restricted”. States belonging to the Guttmacher Institute’s “restrictive” category was renamed “Abortion restricted”. States belonging to the Guttmacher Institute’s “some restrictions/protections” and “protective” categories were renamed “Abortion protected”. Finally, states belonging to the Guttmacher Institute’s “very protective” and “most protective” categories were renamed “Abortion very protected” for the purposes of this study (Table 1). Additionally, we ranked states by maternal mortality rates per 100,000 births based on the most recent report from the Center for Disease Control from 2018 to 2021 [16]. For states with unreportable statistics due to privacy protections, we looked up state specific reports for birth rates from 2018 to 2021 and calculated the maternal mortality rate per 100,000 births [17–20]. States were then assigned one of four maternal mortality groups ranging highest to lowest maternal mortality as follows: “Highest mortality” (26.3–43.5 maternal deaths per 100,000 births), “High mortality 3” (21.7–25.7 maternal deaths per 100,000 births), “Mid-mortality” (16.7–21.2 maternal deaths per 100,000 births), and “Low mortality” (4.8–16.4 maternal deaths per 100,000 births). Of note, 38 states and the District of Columbia currently have MFM programs (See Supplemental materials 3 for a list of all 50 states and District of Columbia that do or do not have a fellowship program and their designations with regards to abortion restrictions and maternal mortality). Geographic regions were defined according to the National Geographic [21].

**Table 1:** States with MFM fellowships and abortion restriction designations

States with MFM fellowship	Guttmacher Institute designation	Renamed group for this study
AL, AR, FL, IN, KY, LA, MO, MS, OK, SC, TN, TX	Most restrictive	Abortion most restrictive
AZ, GA, NC, UT	Very restrictive	
IA, KS, OH, PA, VA, WI	Restrictive	Abortion restrictive
RI	Some restrictions/protections	Abortion protective
CO, CT, DC, HI, IL, MA, MI, WA	Protective	
CA, MD, MN, NJ, NY, NM	Very protective	Abortion most protective
OR, VT	Most protective	

We used Student t-test and ANOVA to calculate unadjusted associations between moral distress and demographic variables, category of abortion restrictions, and category of maternal mortality. Multivariable linear regression was used to examine the association between (1) abortion restrictions and moral distress and (2) maternal mortality and moral distress, adjusting for a priori determined demographic variables (age, gender identity, race/ethnicity, year of training, political identification, and religious identification). Thematic analysis, a well-established research methodology to organize qualitative data into series of themes or patterns [22], was performed for the free text responses elaborating upon moral distress and grouped by thematic elements. The study was approved as exempt by the Institutional Review Board at our institution.

## Results

Of 245 responses (61% response rate), we analyzed 177 complete responses (44% complete response rate). 68 responses were not analyzed due to incomplete nature (most often demographic information was provided, but no scenarios were scored with respect to moral distress). We received at least one response from every state and the District of Columbia with an MFM fellowship other than the state of Arkansas. Most of our respondents identified as female (78.5%), White (71.8%), aged 31–35 years (72.9%), and are training in urban programs (83.1%) that are academic/university-affiliated (92.1%). (Table 2). 37.9% of respondents are training in the Northeast, with the remainder of respondents evenly distributed across the U.S. geographically. Responses were evenly distributed across levels of training, and 12 fellows reported training within a combined program (8 medical genetics, 2 anesthesia/critical care, 1 addiction medicine, and 1 clinical informatics). Most (39.5%) are training at a hospital with an annual delivery volume of 3001–5000 births. 32.8% of our respondents identified as religious and 72.9% reported a political affiliation, with 90.7% of those affiliated with the Democratic party (Table 2).

**Table 2:** Demographics of national sample of maternal-fetal Medicine fellows who responded to the moral distress survey (*n* = 177)

Characteristic	*N* (%)
Gender identity	
Female Male	139 (78.5) 38 (21.5)
Race/ethnicity*	
American Indian or Alaskan Native Asian Black or African American Hispanic White Other Prefer not to answer	1 (0.6) 26 (14.7) 13 (7.3) 13 (7.3) 127 (71.8) 3 (1.7) 3 (1.7)
Age	
26–30 31–35 36–40 41–45 46–50 51–55	12 (6.8) 129 (72.9) 25 (14.1) 6 (3.4) 4 (2.3) 1 (1.0)
Level of training (12 in combined programs)	
PGY-5 PGY-6 PGY-7 PGY-8	50 (28.2) 69 (39.0) 54 (30.5) 4 (2.3)
Region of practice	
Northeast Southeast Midwest Southwest West	67 (37.9) 32 (18.1) 37 (20.9) 17 (9.6) 24 (13.6)
Type of community	
Rural Suburban Urban	5 (2.8) 25 (14.1) 147 (83.1)
Place of practice	
Academic-University based Community nonteaching Community teaching Military	163 (92.1) 1 (1.0) 12 (6.8) 1 (1.0)
Primary inpatient annual delivery volume	
501–1500 1501–3000 3001–5000 5001–8000 8000–10,000 >10,000	6 (3.4) 36 (20.3) 70 (39.5) 47 (26.6) 13 (7.3) 5 (2.8)
Religiosity	
No Yes High Low Prefer not to answer	116 (65.5) 58 (32.8) 32 (55.2) 25 (43.1) 3 (1.7)
Political identification	
No Yes Democratic Libertarian Republican Socialist Other Prefer not to answer	43 (24.3) 129 (72.9) 117 (90.7) 1 (1.0) 6 (4.7) 2 (1.6) 3 (2.3) 5 (2.8)

Data presented as n(%)

*Multiple responses allowed

The mean score for all respondents for the validated portion of the questionnaire was 85.9 *±* 48.8. Female gender identity was associated with higher measures of moral distress on the validated portion of the questionnaire as compared to male gender identity (90.1 *±* 49.2 vs. 70.4 *±* 44.7, *p* < 0.05), whereas more advanced training was associated with higher measures of moral distress on the supplemental questions (20.9 *±* 11.8 vs. 28.5 *±* 15.9 vs. 25.9 *±* 15.6 for PGY-5 vs. PGY-6 vs. PGY-7 and PGY-8 combined, respectively, *p* < 0.05) (Table 3). There was no association between training in states with various levels of abortion restriction or maternal mortality and moral distress on bivariate analysis for either the validated questionnaire or the supplemental questions (Tables 4 and 5). In our multivariable linear regression model examining the association between moral distress and abortion restrictions, higher moral distress on the validated questionnaire was associated with training in a state with increasing abortion restrictions (Beta estimates are all positive when comparing “Abortion most restrictive”, “Abortion restrictive” and “Abortion protective” vs. “Abortion most protective”; beta estimate 27.80 and *p* < 0.01 when comparing association between moral distress and training in a state within “Abortion restrictive” as compared to “Abortion most protective”) (Table 6). Given the supplemental questions are not validated, we did not perform modeling with this subset of the questionnaire. In our multivariable linear regression model examining the association between moral distress and maternal mortality, we did not find any associations.

**Table 3:** Mean scores of moral distress by baseline characteristics

Characteristic	*N* (%)	Validated questions Mean (SD)	Supplemental questions Mean (SD)
Gender identity			
Female	139 (78.5)	90.1 (49.2)*	26.6 (15.7)
Male	38 (21.5)	70.4 (44.7)*	21.6 (11.3)
Race			
^a^Asian	25 (14.1)	92.4 (47.5)	27.6 (15.1)
^a^Black or African American	13 (7.3)	77.5 (38.9)	23.8 (10.8)
^a^White	126 (71.2)	83.8 (49.2)	24.7 (14.9
Other/prefer not to answer (missing)	13 (7.3)	102.0 (56.7)	31.4 (19.1)
Ethnicity			
Hispanic	13 (7.3)	92.5 (49.0)	28.0 (16.3)
Not Hispanic	164 (92.7)	85.3 (48.9)	25.3 (14.9)
Age (years)			
26–35	141 (79.7)	87.2 (50.5)	25.0 (14.3)
36–55	36 (20.3)	80.9 (42.1)	27.6 (17.7)
Training level			
PGY-5	50 (28.2)	76.3 (48.2)	20.9 (11.8)*
PGY-6	69 (39.0)	88.8 (49.9)	28.5 (15.9)*
PGY-7 or PGY-8	58 (32.8)	90.6 (47.8)	25.9 (15.6)*
Region of practice			
Northeast	67 (37.9)	96.8 (52.4)	23.7 (12.7)
Southeast	32 (18.1)	82.5 (53.3)	25.3 (16.8)
Midwest	37 (20.9)	78.7 (36.6)	26.2 (13.8)
Southwest	17 (9.6)	83.9 (39.0)	32.5 (12.7)
West	24 (13.6)	72.4 (52.4)	24.6 (20.6)
Community type			
Suburban/Rural	30 (16.9)	87.8 (50.2)	26.1 (14.6)
Urban	147 (83.1)	76.4 (41.1)	22.7 (16.9)
Place of practice			
Academic-University based	163 (92.1)	86.4 (48.6)	25.5 (15.1)
Other (community, military)	14 (7.9)	79.9 (52.6)	26.1 (14.0)
Primary inpatient annual delivery volume			
5000 or less	106 (59.9)	85.7 (48.4)	24.8 (14.0)
More than 5000	71 (40.1)	86.1 (49.9)	26.5 (16.4)
Identify as religious			
No	116 (65.5)	86.3 (49.7)	24.3 (13.4)
Yes	58 (32.8)	86.6 (46.3)	27.5 (16.9)
Prefer not to answer (missing)	3 (1.7)	-	-
If yes to above, level of religiosity			
High	32 (55.2)	83.0 (46.4)	24.8 (15.6)
Low	25 (43.1)	90.9 (47.7)	31.4 (18.2)
Missing	1 (1.7)	-	-
Identify with political party			
Yes	129 (72.9)	83.4 (46.2)	24.0 (14.1)
Democratic	117 (90.7)	84.5 (47.0)	24.5 (14.4)
Other	12 (9.3)	72.9 (37.8)	19.5 (10.1)
No	43 (24.3)	94.7 (55.7)	28.5 (15.5)
Prefer not to answer (missing)	5 (2.8)	-	-

Numbers may not add up to total N/100% due to structure of the survey

T-tests were used for 2 groups and ANOVA was used for 3 + groups

*significant at *p* < 0.05

^a^Inclusive of those who also identified with Hispanic ethnicity

**Table 4:** Measures of moral distress by categories of abortion restriction

Category	States	*N* (%)	Validated questions Mean (SD)	Supplemental questions Mean (SD)
Abortion most restrictive	AL, AR, AZ, FL, GA, IN, KY, LA, MO, MS, NC, OK, SC, TN, TX, UT	52 (29.4)	75.2 (35.4)	29.2 (15.8)
Abortion restrictive	IA, KS, OH, PA, VA, WI	27 (15.3)	92.6 (57.8)	23.4 (13.6)
Abortion protective	CO, CT, DC, HI, IL, MA, MI, RI, WA	42 (23.7)	98.4 (55.2)	25.1 (12.8)
Abortion most protective	CA, MD, MN, NJ, NM, NY, OR, VT	56 (31.6)	83.2 (48.6)	23.5 (16.2)

No significant differences in scores by abortion restrictions

**Table 5:** Measures of moral distress by categories of maternal mortality

Degree of mortality	States	*N* (%)	Validated questions Mean (SD)	Supplemental questions Mean (SD)
Highest mortality (26.3–43.5 per 100k)	AL, AR, AZ, DC, FL, GA, IN, KY, LA, MS, NC, NM, OK, SC, TN, TX, VA	48 (27.1)	82.0 (45.5)	28.3 (15.7)
High mortality (21.7–25.7)	KS, MO, NJ, NY, OH	49 (27.7)	81.3 (42.9)	24.7 (14.4)
Mid-mortality 2 (16.7–21.2)	CT, HI, IA, IL, MD, MI, PA, RI, WA	43 (24.3)	99.1 (57.7)	25.2 (13.8
Low mortality (4.8–16.4)	CA, CO, MA, MN, OR, UT, VT, WI	37 (20.9)	81.6 (48.4)	23.4 (16.2)

No significant differences in scores by maternal mortality

**Table 6:** Adjusted associations between moral distress on validated questionnaire and demographic variables

	Beta estimate	95% Confidence Interval	*p*-value
White (as compared to non-White)	-1.96	-20.31, 16.39	0.83
Female (as compared to Male)	16.52	-2.10, 35.14	0.08
26–35 years (compared to 36–55 years)	6.60	-12.80, 26.00	0.50
PGY-6 (compared to PGY-5)	12.32	-5.95, 30.58	0.18
PGY-7 and 8 (compared to PGY-5)	10.66	-8.99, 30.30	0.29
Religious (compared to not religious)	3.02	-14.22, 20.25	0.73
Political (as compared to not political)	-12.97	-30.45, 4.50	0.14
Abortion protective*	18.56	-4.43, 41.55	0.11
Abortion restrictive*	**27.80**	**7.05**,** 48.54**	**< 0.01**
Abortion most restrictive*	2.21	-17.79, 22.20	0.83

*As compared to Abortion most protective

34 (19.2%) respondents provided free responses, and thematic analysis revealed several themes. The most commonly referenced theme was around abortion and reproductive justice (22 responses, 64.7%), with the following illustrative quotes:

I feel moral distress all the time for patients who are traveling here to get expensive care and pay out of pocket [for care] that they could have safely had provided locally by perfectly well qualified providers, but cannot get the care they need locally because of state laws and policies that prohibit and deny payment for needed services. It’s appalling.

I work in a Catholic hospital in an abortion restrictive state. I have huge amounts of moral distress because my patients do not have access to contraception in our hospital, and cannot chose a tubal during a C-section for example, or be discharged with LARC placement, and on an on. Then, as an extra layer, the state does not allow abortion care, which is hugely restrictive to my patients, traveling out of state isn’t possible. They need this care and I cannot provide it.

Not being able to offer termination when pregnancy outcomes are poor but maternal life not in danger (ex previable PPROM without evidence of infection).

Other themes included patients not receiving standard of care due to various institutional or provider differences (5 responses, 14.7%), with the following illustrative quote:

Witnessing very disparate quality of care between private MFM office and resident/fellow clinics.

Themes also referenced moral distress as resulting from interdisciplinary power dynamics (3 responses, 8.8%), with the following illustrative quote:

Lack of clear communication between inter- intradisciplinary teams, individualized care instead of teams based care on complex topics, resistance from other teams to accept consult advice.

Another theme surrounded systemic issues involving barriers to payment or other social determinants of health (3 responses, 8.8%), with the following illustrative quote:

Caring for patients whose socioeconomic circumstances significantly impact their care but I cannot improve those circumstances.

Finally, the remaining responses expanding on moral distress emphasized lack of program support (1 response, 2.9%) and medical futility such as in areas of classical cesarean birth at periviable gestational ages (1 response, 2.9%).

## Discussion

In our study of moral distress among MFM fellows, we found that respondents reported an average distress score of 85.9 *±* 48.8, which is on par with previously published scores, such as a score of 96.3 + 54.7 among physician respondents in the study that validated the MMD-HP [1], and female-identifying respondents reported higher measures of moral distress than male-identifying respondents on the validated questions. The association between female gender identity and moral distress has been reported in previous studies [23–25], with speculations regarding varying levels of moral resilience or sensitivity at the root of this finding. In the context of our study, it is possible that female-identifying fellows training in abortion restricted states directly feel the weight of reproductive coercion to a further extent than their male colleagues. Previous studies have also found inconsistent associations between length of training and the perception of moral distress, with some suggesting that the “crescendo effect,” or the buildup of moral distress over time, may disproportionately affect those in training for longer [26]. In the context of this study, it is possible that more senior fellows are more likely to be coordinating care of medically complex individuals at the cusp of medical uncertainty or futility, and may bear the brunt of challenging clinical care, or have had more cumulative exposure to scenarios of moral distress over time.

In bivariate analyses, we did not find significant differences between moral distress and abortion restrictions or maternal mortality. In our multivariable regression model, there was a consistent trend towards more distress among fellows training in states with increasing abortion restrictions, and this difference was significant between those training in “Abortion restrictive” states as compared to “Abortion most protective” states. Recent evidence surrounding moral distress after the Dobbs decision among OB/GYNs support increased moral distress reported by providers in more restrictive states. In a 2023 survey study of 253 abortion providers, those in restrictive states reported higher measures on the moral distress thermometer (which is a visual scale between 0 and 10) as compared to those in protective states [27]. However, in this same study, providers in protective states reported moral distress in the context of caring for those seeking care and having received substandard care from out-of-state with an overburdening of health systems within protective states [28]. It is possible that in our interrelated and increasingly interconnected society, practicing in a silo is a progressively obsolete idea, and policies that impact any patient or provider can have extensive effects, which may dull the observed difference in moral distress between practitioners in restrictive versus protective states. We did not see differential measures of moral distress based on training within states with various levels of maternal mortality. Maternal deaths are fortunately infrequent, and these events may not have reached a clinical threshold to be reflected in perceptions of moral distress amongst trainees. However, there is considerable overlap between states with higher abortion restrictions and maternal mortality, and vice versa (Supplement 3).

An in-depth analysis of the free responses regarding limitations on abortion access and reproductive freedoms further detail specific moral distress as perceived by 19% of respondents who provided qualitative data. Respondents describing feeling held back from being able to offer care they have been trained to provide due to legal and institutional pressures, as well as distress on behalf of patients who incur additional barriers (travel, logistical, financial) in navigating fraught and difficult situations. In addition, providers feel gagged from even discussing the range of options for patients with life-limiting fetal diagnoses or precarious maternal status. Again, it is possible that by utilizing a validated survey tool for this study, we missed out on the opportunity to gear questions to specific challenges within OB/GYN and MFM. However, our supplemental questions and free response field did allow us to capture more nuanced sentiments that could be the basis of the next iteration of surveying regarding moral distress.

Our study has several limitations. First, we were limited by response rate, which introduces significant bias and limits our interpretation of results. Given the voluntary nature of this survey, response bias likely played a significant role in our findings. Our survey was also fairly lengthy, with 33 clinical scenarios, each requesting a level and frequency of distress. We received a total of 245 responses, but 68 survey responses were incomplete and subsequently discarded. The survey itself may benefit from abbreviation and improved specificity as applied to MFM. We considered comparing characteristics between respondents and non-respondents among all MFM fellows to further contextualize our results, but beyond place of training, other demographic characteristics (gender identity, race/ethnicity, etc.) are not assignable without direct questioning. The strength of this study includes the use of a validated survey tool, as well as introduction of additional fields that were both hypothesis generating and allowed for ad lib elaboration on any causes of moral distress within MFM. This allowed us to learn from our colleagues’ particular challenges, both universal and specific to their location of training. Our pool of respondents were fairly representative of the demographics of the MFM fellows nationally and we received geographically diverse responses. We were able to solicit a large number of complete responses, though under our anticipated response rate. Further research could focus on methods to improve this response rate to reduce bias, such as utilizing a more user-friendly and targeted survey tool.

## Conclusion

MFM fellows who identify as female reported higher measures of moral distress, as well as those training in states with more abortion restrictions. Free text responses reveal abortion restrictions to underlie a significant proportion of moral distress. Higher measures of moral distress can lead to physician burnout, compromised patient care, and loss of quality providers, especially in underserved regions. It is especially imperative in our current sociopolitical climate to support physicians directly impacted by legislative restrictions and to find ways of mitigating moral distress in the absence of significant legal change.

## Electronic supplementary material

Below is the link to the electronic supplementary material.

Supplementary Material 1

Supplementary Material 2

Supplementary Material 3

## Data Availability

Data available from the corresponding author on reasonable request.

## Abbreviations

MDS-R: Moral-Distress Survey-Revised
MFM: Maternal-Fetal Medicine
MMD-HP: Measure of Moral Distress – Healthcare Professionals
OB/GYN: Obstetrics and gynecology
