# Supplementary material for: Moral distress among maternal-fetal medicine fellows: a national survey study
Source: BMC Med Ethics. 2025 Feb 28;26:31. doi: 10.1186/s12910-025-01187-4 (PMC11869608; doi:10.1186/s12910-025-01187-4)
Supplement: Supplementary file 1 — Supplementary Material 1 [file 12910_2025_1187_MOESM1_ESM.docx]

**MFM Moral Distress Survey**

**Section 1: Demographics**

1. Do you identify as:
   1. Female
   2. Male
   3. Transgender
   4. Non-binary
   5. Other
   6. Prefer not to answer
2. How would you describe yourself (choose all that apply)
   1. American Indian or Alaskan Native
   2. Asian
   3. Black or African American
   4. Hispanic
   5. Native Hawaiian or Pacific Islander
   6. White
   7. Other
   8. Prefer not to answer
3. What is your age:
   1. <26
   2. 26-30
   3. 31-35
   4. 36-40
   5. 41-45
   6. 46-50
   7. 51-55
   8. 56-60
   9. >60
4. What is your level of training?
   1. PGY5
   2. PGY6
   3. PGY7
   4. PGY8
5. Are you in a combined MFM fellowship program?
   1. Yes (free text option to provide name of additional training)
   2. No
6. How would you characterize your current place of practice?
   1. Academic-University based
   2. Community non-teaching
   3. Community teaching
   4. Military
   5. Other
7. In what state do you practice?
   1. Drop down for all states and DC with MFM fellowship programs
8. In what type of community do you practice currently?
   1. Rural
   2. Suburban
   3. Urban
9. What is your primary inpatient hospital’s annual delivery volume?
   1. <500
   2. 501-1500
   3. 1501-3000
   4. 3001-5000
   5. 5001-8000
   6. 8001-10000
   7. >10000
10. Do you identify as religious?
    1. Yes
       1. What is your level of religiosity
          1. Low
          2. High
    2. No
    3. Prefer not to answer
11. Do you identify with a political party
    1. Yes
       1. Democratic
       2. Libertarian
       3. Republican
       4. Socialist
       5. Other (free text)
    2. No
    3. Prefer not to answer

# Measure of Moral Distress – Healthcare Professionals (MMD-HP)

**Moral distress occurs when professionals cannot carry out what they believe to be ethically appropriate actions because of constraints or barriers. This survey lists situations that occur in clinical practice. If you have experienced these situations they may or may not have been morally distressing to you. Please indicate how frequently you have experienced each item. Also, rank how distressing these situations are for you. If you have never experienced a particular situation, select “0” (never) for frequency. Even if you have not experienced a situation, please indicate how distressed you would be if it occurred in your practice. Note that you will respond to each item by checking the appropriate column for two dimensions: *Frequency* and *Level of Distress*.**

**­­­­**

|  | **Frequency** | | | | | | **Level of Distress** | | | | |
| --- | --- | --- | --- | --- | --- | --- | --- | --- | --- | --- | --- |
|  | Never Very  frequently | | | | | | None Very  distressing | | | | |
|  | 0 | 1 | 2 | 3 | 4 | 0 | | 1 | 2 | 3 | 4 |
| 1. Witness healthcare providers giving “false hope” to a patient or family. |  |  |  |  |  |  | |  |  |  |  |
| 1. Follow the family’s insistence to continue aggressive treatment even though I believe it is not in the best interest of the patient. |  |  |  |  |  |  | |  |  |  |  |
| 1. Feel pressured to order or carry out orders for what I consider to be unnecessary or inappropriate tests and treatments. |  |  |  |  |  |  | |  |  |  |  |
| 1. Be unable to provide optimal care due to pressures from administrators or insurers to reduce costs. |  |  |  |  |  |  | |  |  |  |  |
| 1. Continue to provide aggressive treatment for a person who is most likely to die regardless of this treatment when no one will make a decision to withdraw it. |  |  |  |  |  |  | |  |  |  |  |
| 1. Be pressured to avoid taking action when I learn that a physician, nurse, or other team colleague has made a medical error and does not report it. |  |  |  |  |  |  | |  |  |  |  |
| 1. Be required to care for patients whom I do not feel qualified to care for. |  |  |  |  |  |  | |  |  |  |  |
| 1. Participate in care that causes unnecessary suffering or does not adequately relieve pain or symptoms. |  |  |  |  |  |  | |  |  |  |  |
| 1. Watch patient care suffer because of a lack of provider continuity. |  |  |  |  |  |  | |  |  |  |  |
| 1. Follow a physician’s or family member’s request not to discuss the patient’s prognosis with the patient/family. |  |  |  |  |  |  | |  |  |  |  |
| 1. Witness a violation of a standard of practice or a code of ethics and not feel sufficiently supported to report the violation. |  |  |  |  |  |  | |  |  |  |  |
| 1. Participate in care that I do not agree with, but do so because of fears of litigation. |  |  |  |  |  |  | |  |  |  |  |
| 1. Be required to work with other healthcare team members who are not as competent as patient care requires. |  |  |  |  |  |  | |  |  |  |  |
| 1. Witness low quality of patient care due to poor team communication. |  |  |  |  |  |  | |  |  |  |  |
| 1. Feel pressured to ignore situations in which patients have not been given adequate information to ensure informed consent. |  |  |  |  |  |  | |  |  |  |  |
| 1. Be required to care for more patients than I can safely care for. |  |  |  |  |  |  | |  |  |  |  |
| 1. Experience compromised patient care due to lack of resources/equipment/bed capacity. |  |  |  |  |  |  | |  |  |  |  |
| 1. Experience lack of administrative action or support for a problem that is compromising patient care. |  |  |  |  |  |  | |  |  |  |  |
| 1. Have excessive documentation requirements that compromise patient care. |  |  |  |  |  |  | |  |  |  |  |
| 1. Fear retribution if I speak up. |  |  |  |  |  |  | |  |  |  |  |
| 1. Feel unsafe/bullied amongst my own colleagues. |  |  |  |  |  |  | |  |  |  |  |
| 1. Be required to work with abusive patients/family members who are compromising quality of care. |  |  |  |  |  |  | |  |  |  |  |
| 1. Feel required to overemphasize tasks and productivity or quality measures at the expense of patient care. |  |  |  |  |  |  | |  |  |  |  |
| 1. Be required to care for patients who have unclear or inconsistent treatment plans or who lack goals of care. |  |  |  |  |  |  | |  |  |  |  |
| 1. Work within power hierarchies in teams, units, and my institution that compromise patient care. |  |  |  |  |  |  | |  |  |  |  |
| 1. Participate on a team that gives inconsistent messages to a patient/family. |  |  |  |  |  |  | |  |  |  |  |
| 1. Work with team members who do not treat vulnerable or stigmatized patients with dignity and respect. |  |  |  |  |  |  | |  |  |  |  |
