# Supplementary material for: Moral distress among maternal-fetal medicine fellows: a national survey study
Source: BMC Med Ethics. 2025 Feb 28;26:31. doi: 10.1186/s12910-025-01187-4 (PMC11869608; doi:10.1186/s12910-025-01187-4)
Supplement: Supplementary file 2 — Supplementary Material 2 [file 12910_2025_1187_MOESM2_ESM.docx]

|  | **Frequency** | | | | | | **Level of Distress** | | | | |
| --- | --- | --- | --- | --- | --- | --- | --- | --- | --- | --- | --- |
|  | Never Very  frequently | | | | | | None Very  distressing | | | | |
|  | 0 | 1 | 2 | 3 | 4 | 0 | | 1 | 2 | 3 | 4 |
| 1. Caring for patients requiring substantial balancing of maternal risk with fetal benefit |  |  |  |  |  |  | |  |  |  |  |
| 1. Caring for patients when there is significant medical uncertainty regarding prognosis or standard of care |  |  |  |  |  |  | |  |  |  |  |
| 1. Caring for high-risk maternal patients with expected post-natal demise or other situations of medical futility |  |  |  |  |  |  | |  |  |  |  |
| 1. Working with limited resources (ie time, staff) when certain patients require intense amount of resources |  |  |  |  |  |  | |  |  |  |  |
| 1. Caring for patients desiring pregnancy termination |  |  |  |  |  |  | |  |  |  |  |
| 1. Transferring patients for services not available at your institution |  |  |  |  |  |  | |  |  |  |  |

If there are other situations in which you have felt moral distress, please write and score them here:
