# Supplementary material for: Moral distress among maternal-fetal medicine fellows: a national survey study
Source: BMC Med Ethics. 2025 Feb 28;26:31. doi: 10.1186/s12910-025-01187-4 (PMC11869608; doi:10.1186/s12910-025-01187-4)
Supplement: Supplementary file 3 — Supplementary Material 3 [file 12910_2025_1187_MOESM3_ESM.docx]

| State or District | Two-letter abbr. | Fellowship | Guttmacher designation | Renamed Abortion group | Maternal mortality rate* | Mortality group |
| --- | --- | --- | --- | --- | --- | --- |
| ALABAMA | AL | Yes | Most restrictive | Abortion most restrictive | 41.4 | Highest mortality |
| ALASKA | AK | No |  |  |  |  |
| ARIZONA | AZ | Yes | Very restrictive | Abortion most restrictive | 31.4 | Highest mortality |
| ARKANSAS | AR | Yes | Most restrictive | Abortion most restrictive | 43.5 | Highest mortality |
| CALIFORNIA | CA | Yes | Very protective | Abortion most protective | 10.1 | Low mortality |
| COLORADO | CO | Yes | Protective | Abortion protective | 15.2 | Low mortality |
| CONNECTICUT | CT | Yes | Protective | Abortion protective | 16.7 | Mid-mortality |
| DELAWARE | DE | No |  |  |  |  |
| DISTRICT OF COLUMBIA | DC | Yes | Protective | Abortion protective | 30.7 | High mortality |
| FLORIDA | FL | Yes | Most restrictive | Abortion most restrictive | 26.3 | Highest mortality |
| GEORGIA | GA | Yes | Very restrictive | Abortion most restrictive | 33.9 | Highest mortality |
| HAWAII | HI | Yes | Protective | Abortion protective | 18.4 | Mid-mortality |
| IDAHO | ID | No |  |  |  |  |
| ILLINOIS | IL | Yes | Protective | Abortion protective | 17.3 | Mid-mortality |
| INDIANA | IN | Yes | Most restrictive | Abortion most restrictive | 31.1 | Highest mortality |
| IOWA | IA | Yes | Restrictive | Abortion restrictive | 20.2 | Mid-mortality |
| KANSAS | KS | Yes | Restrictive | Abortion restrictive | 22.0 | High mortality |
| KENTUCKY | KY | Yes | Most restrictive | Abortion most restrictive | 38.4 | Highest mortality |
| LOUISIANA | LA | Yes | Most restrictive | Abortion most restrictive | 39.0 | Highest mortality |
| MAINE | ME | No |  |  |  |  |
| MARYLAND | MD | Yes | Very protective | Abortion most protective | 21.2 | Mid-mortality |
| MASSACHUSETTS | MA | Yes | Protective | Abortion protective | 15.3 | Low mortality |
| MICHIGAN | MI | Yes | Protective | Abortion protective | 19.4 | Mid-mortality |
| MINNESOTA | MN | Yes | Very protective | Abortion most protective | 12.6 | Low mortality |
| MISSISSIPPI | MS | Yes | Most restrictive | Abortion most restrictive | 43.0 | Highest mortality |
| MISSOURI | MO | Yes | Most restrictive | Abortion most restrictive | 25.7 | High mortality |
| MONTANA | MT | No |  |  |  |  |
| NEBRASKA | NE | No |  |  |  |  |
| NEVADA | NV | No |  |  |  |  |
| NEW HAMPSHIRE | NH | No |  |  |  |  |
| NEW JERSEY | NJ | Yes | Very protective | Abortion most protective | 25.7 | High mortality |
| NEW MEXICO | NM | Yes | Very protective | Abortion most protective | 30.2 | Highest mortality |
| NEW YORK | NY | Yes | Very protective | Abortion most protective | 21.7 | High mortality |
| NORTH CAROLINA | NC | Yes | Very restrictive | Abortion most restrictive | 26.5 | Highest mortality |
| NORTH DAKOTA | ND | No |  |  |  |  |
| OHIO | OH | Yes | Restrictive | Abortion restrictive | 23.8 | High mortality |
| OKLAHOMA | OK | Yes | Most restrictive | Abortion most restrictive | 30.3 | Highest mortality |
| OREGON | OR | Yes | Most protective | Abortion most protective | 16.4 | Low mortality |
| PENNSYLVANIA | PA | Yes | Restrictive | Abortion restrictive | 16.7 | Mid-mortality |
| RHODE ISLAND | RI | Yes | Some protections/ restrictions | Abortion protective | 17.0 | Mid-mortality |
| SOUTH CAROLINA | SC | Yes | Most restrictive | Abortion most restrictive | 32.7 | Highest mortality |
| SOUTH DAKOTA | SD | No |  |  |  |  |
| TENNESSEE | TN | Yes | Most restrictive | Abortion most restrictive | 41.7 | Highest mortality |
| TEXAS | TX | Yes | Most restrictive | Abortion most restrictive | 28.1 | Highest mortality |
| UTAH | UT | Yes | Very restrictive | Abortion most restrictive | 16.1 | Low mortality |
| VERMONT | VT | Yes | Most protective | Abortion most protective | 4.8 | Low mortality |
| VIRGINIA | VA | Yes | Restrictive | Abortion restrictive | 29.1 | Highest mortality |
| WASHINGTON | WA | Yes | Protective | Abortion protective | 20.4 | Mid-mortality |
| WEST VIRGINIA | WV | No |  |  |  |  |
| WISCONSIN | WI | Yes | Restrictive | Abortion restrictive | 11.6 | Low mortality |
| WYOMING | WY | No |  |  |  |  |

*per 100,000 births
